# Evaluating the effect of injection protocols on intrathecal solute dispersion in non-human primates: an in vitro study using a cynomolgus cerebrospinal fluid system

**DOI:** 10.1186/s12987-024-00556-2

**Published:** 2024-07-26

**Authors:** Goutham Kumar Reddy Burla, Dev Shrestha, Mayumi Bowen, Joshua D. Horvath, Bryn A. Martin

**Affiliations:** 1 Department of Chemical and Biological Engineering, University of Idaho, 875 Perimeter Dr. MC1122, Moscow, ID 83844 USA; 2 Genentech, Inc., a member of the Roche Group, South San Francisco, CA USA; 3 Alcyone Therapeutics Inc., Lowell, MA USA; 4 Flux Neuroscience, LLC., Troy, ID USA

## Abstract

**Background:**

Achieving effective drug delivery to the central nervous system (CNS) remains a challenge for treating neurological disorders. Intrathecal (IT) delivery, which involves direct injection into the cerebrospinal fluid (CSF), presents a promising strategy. Large animal studies are important to assess the safety and efficacy of most drugs and treatments and translate the data to humans. An understanding of the influence of IT injection parameters on solute distribution within the CNS is essential to optimize preclinical research, which would potentially help design human clinical studies.

**Methods:**

A three-dimensional (3D) in vitro model of a cynomolgus monkey, based on MRI data, was developed to evaluate the impact of lumbar injection parameters on intrathecal solute dispersion. The parameters evaluated were (a) injection location, (b) bolus volume, (c) flush volume, (d) bolus rate, and (e) flush rate. To simulate the CSF flow within the subarachnoid space (SAS), an idealized CSF flow waveform with both cardiac and respiratory-induced components was input into the model. A solution of fluorescein drug surrogate tracer was administered in the lumbar region of the 3D in vitro model filled with deionized water. After injection of the tracer, the CSF system wide-solute dispersion was imaged using high-resolution cameras every thirty seconds for a duration of three hours. To ensure repeatability each injection protocol was repeated three times. For each protocol, the average spatial–temporal distribution over three hours post-injection, the area under the curve (AUC), and the percent injected dose (%ID) to extra-axial CSF (eaCSF) at three hours were determined.

**Results:**

The changes to the lumbar injection parameters led to variations in solute distribution along the neuro-axis. Specifically, injection location showed the most impact, enhancing the delivery to the eaCSF up to + 10.5%ID (p = 0.0282) at three hours post-injection. Adding a post-injection flush of 1.5 ml at 1 ml/min increased the solute delivery to the eaCSF by + 6.5%ID (p = 0.0218), while the larger bolus volume resulted in a + 2.3%ID (p = 0.1910) increase. The bolus and flush rates analyzed had minimal, statistically non-significant effects.

**Conclusion:**

These results predict the effects of lumbar injection parameters on solute distribution in the intrathecal space in NHPs. Specifically, the choice of injection location, flush, and bolus volume significantly improved solute delivery to eaCSF. The in vitro NHP CSF model and results offer a system to help predict and optimize IT delivery protocols for pre-clinical NHP studies.

## Introduction

The blood–brain barrier (BBB) controls the exchange of molecules between the blood and brain tissue to maintain an optimal chemical environment in the central nervous system (CNS). While it selectively allows essential molecules to pass through, it blocks nearly all large molecules and over 98% of small molecules [1], making it challenging to administer therapeutics to the CNS via the bloodstream. A promising alternative to this challenge is intrathecal (IT) delivery, where drugs are injected into the thecal sac containing cerebrospinal fluid (CSF). The CSF bathes the brain and spinal cord, providing a more direct pathway that bypasses the BBB, allowing for potentially more efficient CNS distribution while limiting the risk of off-target drug exposure and potential toxicity. According to the classical hypothesis [2–5], the CSF is produced within the choroid plexus and circulates through various regions, such as the ventricles, cisterns, and spinal subarachnoid space (SAS) with a complex pattern. Eventually, the CSF is believed to be absorbed into the blood primarily via the arachnoid villi or granulations at the superior sagittal sinus. A new hypothesis suggests that CSF is produced and absorbed throughout the CSF system via filtration and reabsorption of water through capillary walls into surrounding CNS tissues [6]. Further research is required to understand the sources of CSF production and absorption. The flow of the CSF is oscillatory, meaning it has a rhythmic movement, with nearly zero net flow, that moves in synchrony with the cardiac and respiratory cycles [7]. Once a solute is injected into the CSF, it disperses along the spinal subarachnoid space (SAS) and around the brain while being absorbed into the CNS tissue. Depending on the size of the particles, the solute may leak out of the CSF system via many complex routes including nerve root sleeves, cribriform plate, dural lymphatics, and potentially other pathways [8–10]. In recent years, there has been increasing interest in the use of IT delivery of drugs to treat a range of CNS disorders, including chronic pain, leptomeningeal cancer and glioblastoma, multiple sclerosis, and neurodegenerative diseases including amyotrophic lateral sclerosis, Huntington’s disease, spinal muscular atrophy, and many others. As of 2023, there have been more than 345 clinical trials utilizing intrathecal injections [11]. Thus, understanding solute distribution in the IT space is vital for developing new therapies and improving existing delivery strategies.

In pre-clinical studies of most drugs and treatments, large animals are used to study how the drug behaves and to assess the safety and efficacy before human clinical trials. Nonhuman primates (NHP) are preferred because they have similar genes and proteins as those in humans, resulting in similar immunity and metabolism [12–16]. Additionally, their CNS system and upright spine orientation closely resemble humans, and have been used in toxicology studies of different intrathecal drugs, such as antisense oligonucleotide (ASO) [17] and adeno-associated viruses (AAV) [18, 19]. Various non-invasive imaging techniques such as positron emission tomography (PET) and real-time MRI were utilized in NHP studies to investigate IT enzyme replacement therapies [20, 21], distribution of phage particles [22], the kinetics of AAV vector [23] and the impact of convective IT infusion algorithms [24].

While these studies provide crucial insights into the CNS distribution of various therapeutics, animal experimentation in NHPs is limited due to ethical and economic constraints. A cost-effective approach to understanding IT solute dispersion is to utilize in silico and in vitro models. They have been instrumental in testing different injection scenarios while also providing valuable insights into the complex dynamics of CSF. Furthermore, these techniques allow for continuous monitoring of solute movement after injection, which is not feasible with in vivo imaging studies. Several physical and computational models representing the CSF system of humans were employed to investigate various factors contributing to IT dispersion. For example, Hettiarachchi et al. [25] conducted infusion experiments using radionucleotide and fluorescent dye in a tube representing the human spinal canal. Tracer distribution was studied under stagnant and pulsatile flow conditions, and patient-specific geometry was considered in CFD predictions. Hsu et al. [26] evaluated the effect of frequency and magnitude of CSF oscillations on drug distribution using a CFD model. Tangen et al. [27] used CFD models and investigated the drug distribution by varying infusion settings, drug type, and CSF flow conditions. Khani et al. [28] quantified the impact of nerve roots on CSF dynamics by using an anatomically realistic CFD model. In a later study, Khani et al. [29] investigated the influence of respiration, heart rate, and infusion settings on IT delivery. While these studies sought to represent humans, Tangen et al. [30] utilized PET imaging and CFD models to investigate CNS distribution in cynomolgus monkeys. The injection scenarios were tested computationally using idealized models without the intracranial space.

Despite the outstanding contributions, we have relatively little quantitative information about the impact of IT injection parameters on intrathecal solute dispersion in NHPs. Understanding the distribution in NHPs is essential for optimizing drug delivery protocols during the pre-clinical stages and successfully scaling them to the clinical stage. The present study aims to (a) develop an anatomically realistic 3D CSF system of a cynomolgus monkey, (b) utilize the model to conduct repeatable in-vitro lumbar injections with a range of lumbar puncture-based injection volumes, rates, and locations, and (c) quantify the impact of these injection parameters on delivery to extra-axial CSF (eaCSF). To our knowledge, this is the first 3D cynomolgus in vitro model used to investigate the effects of injection parameters on IT drug delivery. This physical model, bridges the gap between existing human models and the commonly employed species for translational studies, providing a cost-effective platform for rapid prototyping delivery devices and conducting comparative analysis across species. Our results provide quantitative predictions into how specific lumbar puncture-based injection protocols may impact CSF system solute dispersion to the brain.

## Methods

The overall approach was to examine the effect of injection parameters on the distribution of fluorescein tracer in the CSF system after lumbar injection using a subject-specific 3D cynomolgus monkey in vitro model. The parameters under investigation were: (a) injection location, (b) bolus volume, (c) flush volume, (d) bolus rate, and (e) flush rate (Table 1). A solution of fluorescein diluted in deionized water was intrathecally injected by a lumbar puncture needle into the lumbar region of the in vitro model filled with deionized water. To visualize the tracer dispersion within the CSF, the model was imaged using high-resolution cameras at thirty-second intervals for a duration of three hours. The dosage of the tracer and lumbar injection needle geometry remained constant across all experiments.

**Table 1:** List of experiments

Experiment	Location	Tracer concentration (μM)	Bolus volume (mL)	Bolus rate (mL/min)	Flush volume (mL)	Flush rate (mL/min)
2NHP2	L4/L5	331	0.5	1	1.5	1
3G1	L3/L4	331	0.5	1	1.5	1
1NHP1	L3/L4	165.5	1	1	–	–
3G2	L3/L4	82.75	2	1	–	–
1NHP3	L4/L5	165.5	1	0.1	–	–
1NHP2	L4/L5	165.5	1	1	–	–
2G2	L4/L5	165.5	1	1	1.5	1
5NHP1	L4/L5	165.5	1	1	1.5	0.5

### CSF geometry and waveform

**Fig. 1:**
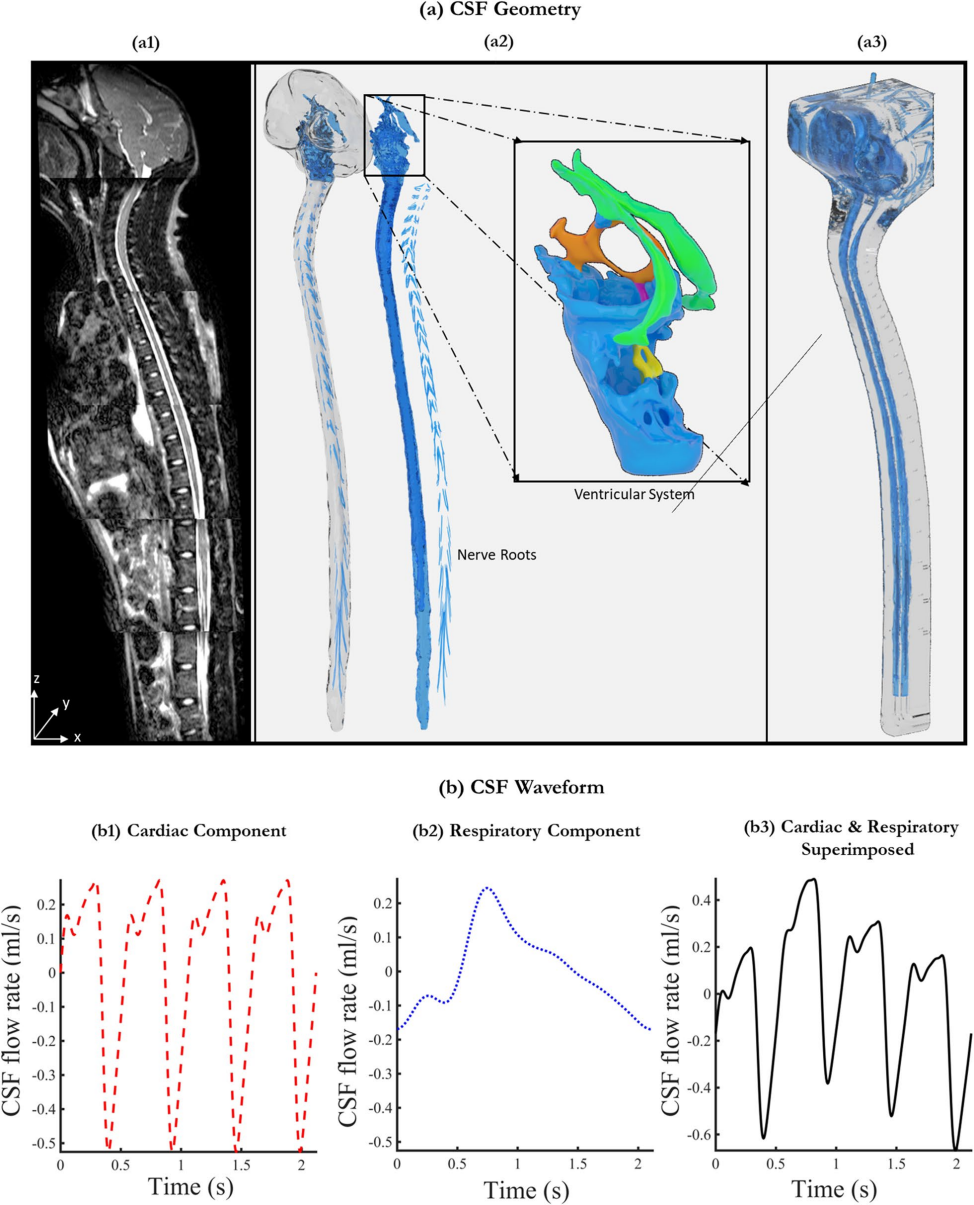
Overview of CSF geometry and waveform. **a1** Mid-sagittal T2-weighted MR image of NHP. **a2** Manually segmented CSF spaces obtained from MRI scans including idealized ventricular system and nerve roots. **a3** Visualization of the 3D printed model. **b1** Plot showing CSF waveform measured at C2-C3 using cardiac gated PC-MRI. **b2** The derived respiratory component from measurements in humans. **b3** The final waveform used as input is obtained by superimposing both cardiac and respiration components

The 3D model utilized in this study was constructed based on MRI measurements obtained from a study by Khani et al. [31] on a 5 year-old adult cynomolgus monkey. Utilizing the VISTA protocol, Khani et al. acquired a series of high-resolution axial T2-weighted MR images (Fig. 1.a1). The CSF space was manually segmented from these images using ITK-SNAP (Version 3.4.0, University of Pennsylvania, USA). Following this segmentation, nerve rootlets were incorporated into the model along the spinal cord at all vertebral levels (Fig. 1.a2). The resulting model, which had a CSF volume of 16.86 ml, was exported in the.STL format for stereolithography-based printing (Fig. 1.a3). To complete the model, injection ports were added to the printed model at L4/L5 and L3/L4 levels. The 3D-printed model was micro-CT scanned, with an isotropic voxel size of 101.6 microns, in three sections to verify the CSF geometry. MATLAB was used to process the axial slices, segment the CSF space, and calculate the regional volumes in the 3D-printed model at 1 mm slice thickness intervals.

Khani et al. [31], measured CSF flow using cardiac-gated phase-contrast MRI (PC-MRI) at six distinct locations along the spines of eight monkeys. The average waveform quantified at the C2-C3 level was used as the cardiac component in our study (Fig. 1.b1). Due to the lack of data on CSF oscillations caused by respiration in NHPs, the respiratory component of the CSF flow was extrapolated based on measurements obtained in humans. During natural breathing, the respiratory flow waveform, as described by Yildiz et al. [7] was employed to determine the respiration component (Fig. 1.b2). We scaled the magnitude and frequency of this waveform to be 0.52 and 4.29 of the cardiac components, respectively. The number 0.52 represents the flow ratio, which is calculated as the ratio of flow amplitudes of the respiratory and the cardiac components under natural breathing conditions measured by Yildiz et al. [7]. The number 4.29 is the average heart rate to respiration rate ratio in cynomolgus monkeys [32–35]. Finally, both components were superimposed to derive a combined CSF waveform with a heart rate of 120 bpm, respiration rate of ~ 28 breaths per minute, and stroke volume of 0.2597 ml (Fig. 1.b3).

### Experimental setup

**Fig. 2:**
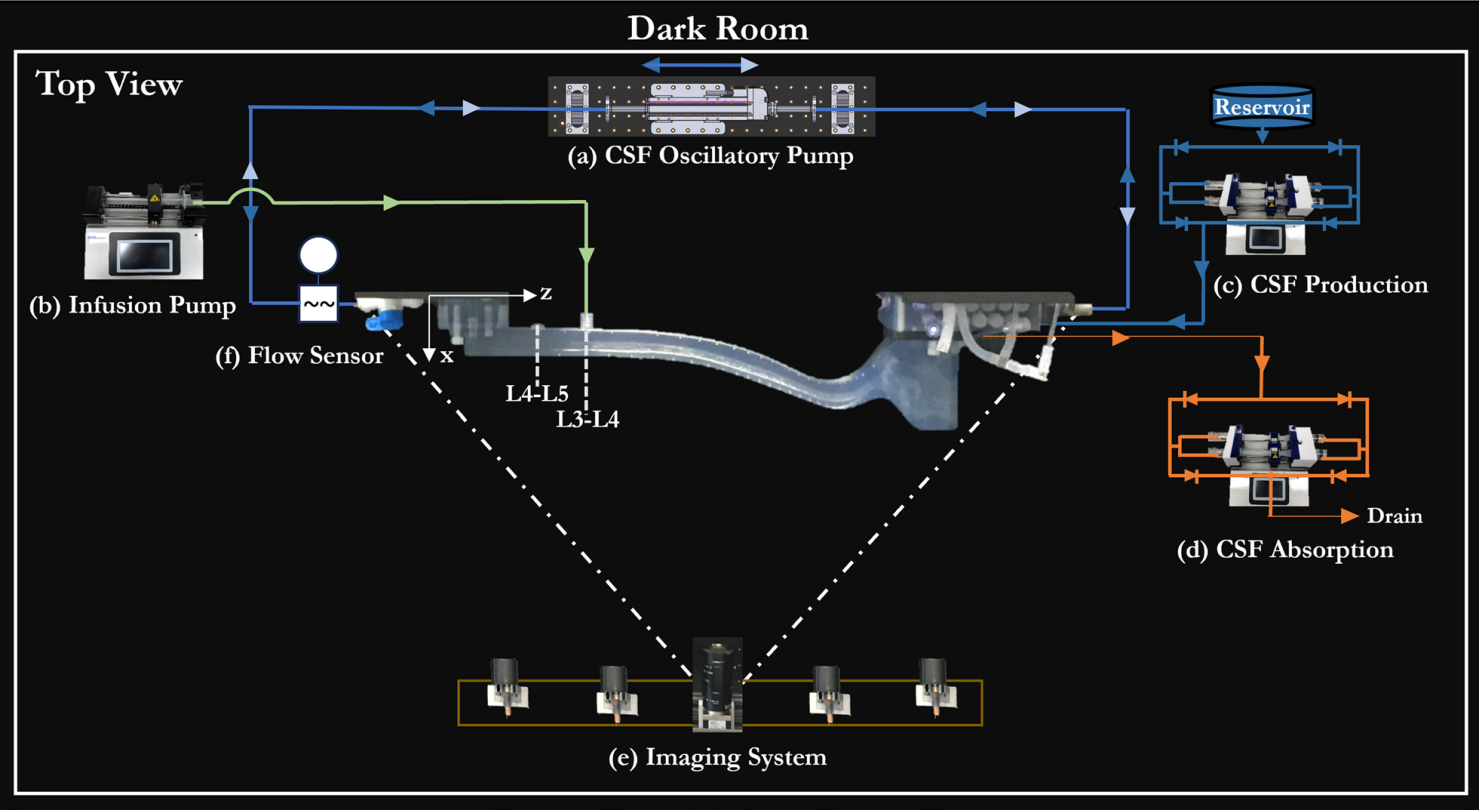
The top view schematic of the test setup in a dark room. **a** A servo tube actuator is connected to the 3D model to generate CSF oscillatory flow. **b** The infusion pump injects the tracer at the corresponding flow rates and volumes, as listed in Table 1. **c** The CSF production pump withdraws deionized water from the reservoir and infuses it into the ventricles at 0.018 ml/min. **d** The CSF absorption pump drains the water from the intracranial space at 0.018 ml/min. **e** The Imaging system consists of a blue LED array and two cameras positioned to capture the model with the same field of view but with different exposure settings. **f** Transonic flow sensor measuring the inlet flow waveform generated by the oscillatory pump

The overall experimental setup was previously described by Seiner et al. [36] using a human model. In brief, all the experiments were conducted in a 1 m^3^ dark room with the model mounted in a left lateral recumbent position. In the model, oscillatory flow was generated using a bi-directional servo tube actuator (Fig. 2.a), connected to both the caudal and cranial ends by custom fittings. The actuator was programmed to produce the derived CSF flow waveform shown in Fig. 1.b3. The actual CSF flow was verified by employing a transonic flow meter (Transonic, T402) and an in-line sensor (Transonic, 4PXN) at an inlet located at the caudal end (Fig. 2.f). Fluorescein tracer was injected posteriorly using a dual syringe pump via a 25G needle at the pre-determined injection location (Fig. 2.b). Additionally, to represent CSF production and absorption, separate dual syringe pumps were utilized. The production pump (Fig. 2.c) continuously withdrew DI water from the reservoir and infused it into the CSF space, through the ports in lateral ventricles at a rate of 0.018 ml/min. The specified production rate corresponds to the mean clearance of inulin observed in NHPs following intraventricular injections [37]. Simultaneously, the absorption pump (Fig. 2.d) drew DI water from the intracranial space, representing absorption, also at the rate of 0.018 ml/min. To ensure accuracy, all dual syringe pumps (Fig. 2.b,c,d) were calibrated by collecting and measuring pump outflow over a known period. The imaging system consists of two cameras that have the same field of view and an array of blue LED sources (Fig. 2.d). To increase the dynamic range of the imaging system, both cameras had different exposure settings. The exposure settings of each camera are individually optimized to avoid over-saturation. The images were acquired at 2168 × 4096 resolution in 30 s intervals for a duration of three hours.

### Quantification of tracer concentration, %ID, and AUC

The axial distribution of the tracer was quantified by adapting the procedures previously described by Seiner et al. [36]. In brief, the average pixel intensity along the z-axis was calculated for both the low- and high-exposure images. These individual datasets were subsequently converted to molar concentrations of the tracer by using the calibration maps created for each camera. To merge the datasets, we selected the data points < 10 µM in the low-exposure set and replaced them with the corresponding points from the high-exposure set. The 10 µM threshold was chosen as it is the mid-point across the calibration maps. This merged data was used to generate spatial–temporal plots and area under the curve (AUC) profiles. The trapezoidal integration values over one hour and three hours post-injection are termed AUC_0–1 h_ and AUC_0–3 h_, respectively.

To quantify the influence of injection parameters after three hours, we computed the delivery of the tracer as a percent injected dose (%ID) to eaCSF by using Eq. (1). In the equation, $V\left(z\right)$ is the CSF volume at a slice-z and $C\left(z\right)$ is the concentration of tracer at that slice. The range for z is from 0 to 60 mm, spanning from the foramen magnum to the top of the model.

1 $$\%ID to eaCSF=\left(\frac{\sum_{z=0}^{z= 60}V\left(z\right)\times C(z) }{ Injected mass}\right)\times 100$$

### Repeatability and statistical analysis

All experiments in this study are shown in Table 1. Each experiment was repeated three times, and the standard deviation of tracer concentration at each z-location and time points post injection was visualized as a spatial–temporal plot. Bland–Altman plots were generated for each injection protocol to quantify the mean difference and limits of agreement between the repetitions. The 95% confidence intervals of the mean of the repetitions were calculated and represented as a percent of dynamic range (%DR) by using Eq. (3), where ${C}_{max}$ is the maximum concentration.

2 $$\%DR= \frac{95\% CI}{{C}_{max}}\times 100$$

The eight experiments are grouped into five injection parameter groups (a) Injection location (2NHP2-3G1), (b) Bolus volume (1NHP1-3G2), (c) Bolus rate (1NHP3-1NHP2), (d) Flush volume (1NHP2-2G2) and (e) Flush rate (5NHP1-2G2). The change in %ID to eaCSF (Δ%ID) was determined within each group to quantify the parametric impact of the variable. An unpaired t-test was conducted for each group to understand the statistical differences at a significance level of α = 0.05.

## Results

### Verification of boundary conditions and repeatability of experiments

**Fig. 3:**
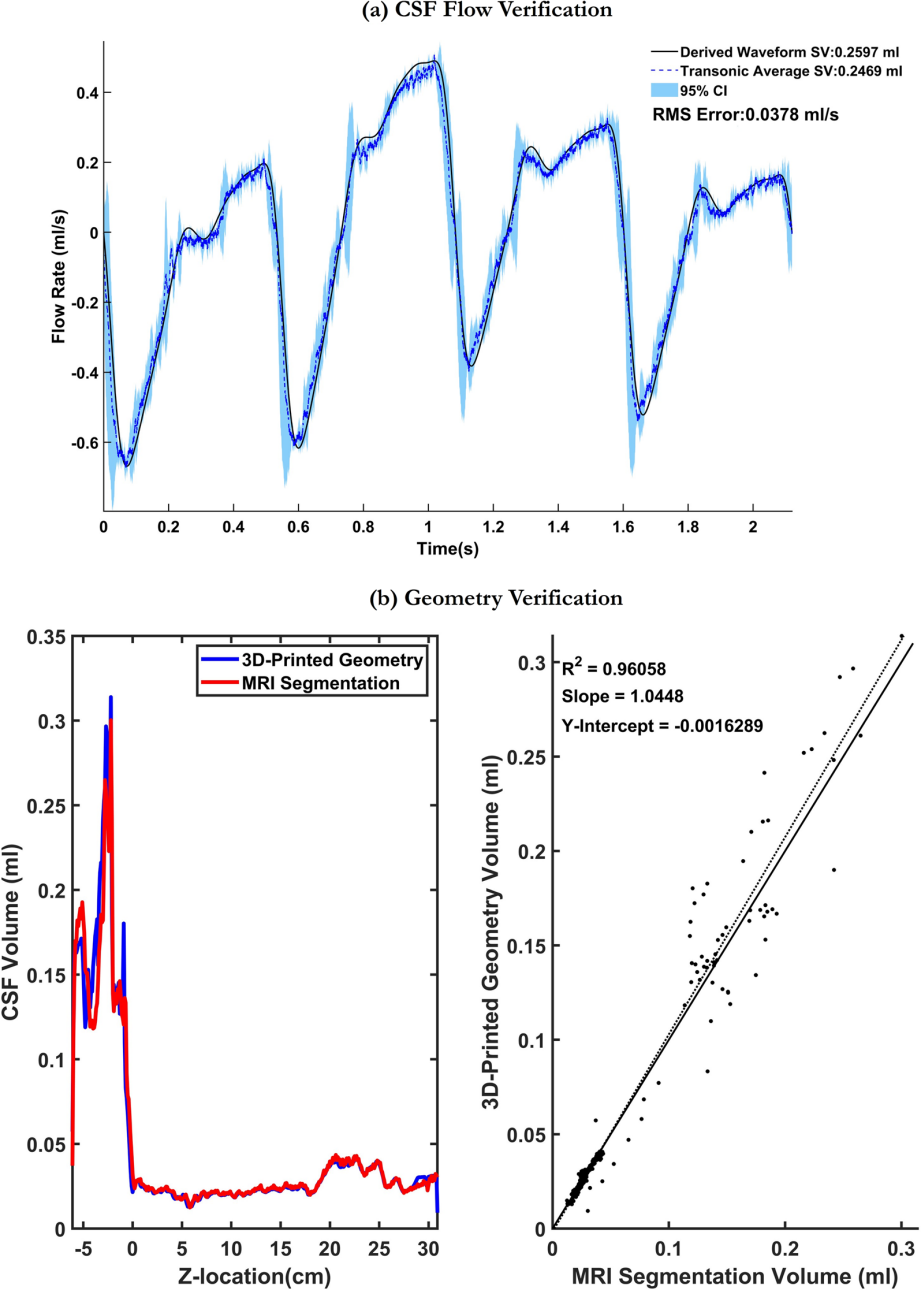
Verification of geometric and flow boundary conditions. **a** Comparison of derived waveform with the average transonic flow meter readings obtained at the caudal end of the flow model with 95% confidence intervals calculated based on five flow cycle repetitions. **b** Comparison of CSF volume measurements obtained from the segmented MRI data to the CSF volume measurements obtained from micro-CT scans collected on the 3D-printed model

**Fig. 4:**
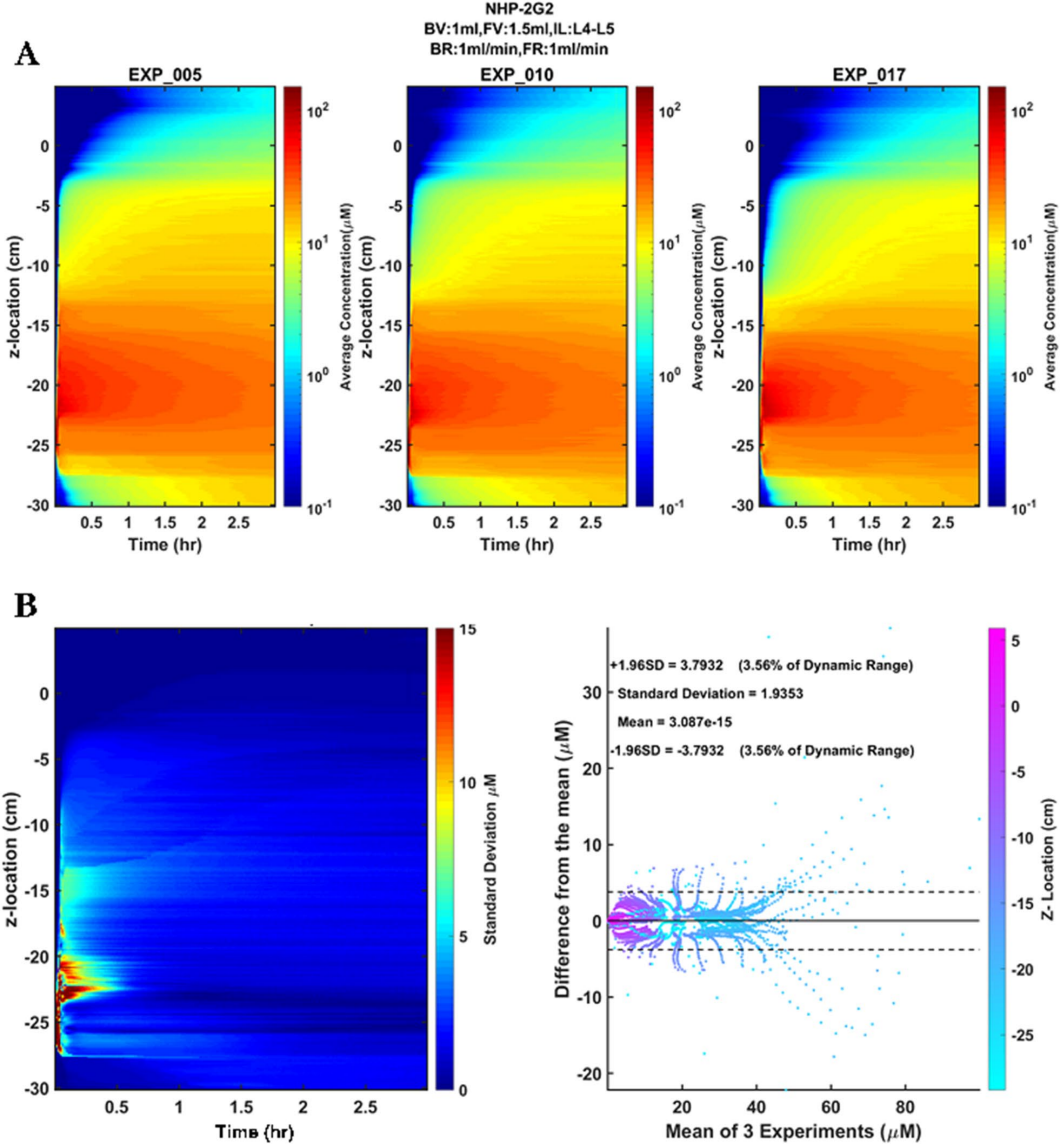
Repeatability and reliability of an experiment. **a** The spatial–temporal plots of each repetition (2G2 case shown here). **b** The plot shows the standard deviation of repetitions at each location and time point, and the Bland–Altman plot shows the difference between each repetition from the mean of repetitions. The dotted lines indicate the 95% confidence intervals

Figure 3a compares the average of five transonic flowmeter measurements obtained at the inlet of the spine to the input waveform. The plot demonstrates that the custom-made oscillatory pump accurately reproduced the desired flow at the inlet (RMSE = 0.0378 ml/s). The axial slice volume measurements, obtained from the CT scans, were compared to the CSF volume obtained through MRI measurements at 1 mm slice thickness (Fig. 3b). Total model volume for the 3D MRI segmentation was 16.86 mL compared to 17.44 mL for the 3D printed model. Correlation of 3D MRI segmentation with 3D printed model segmentation from micro-CT showed strong agreement (R^2^ = 0.96, slope = 1.04, y-intercept = − 0.0016). The repeatability of the 2G2 experiment is shown in Fig. 4a. Variations in tracer distribution across repetitions are mainly noticed at the injection location (Fig. 4b). The standard deviation and 95% CI of all experiments conducted in this study are listed in Table 2. Overall, strong repeatability between experiments was observed. The maximum standard deviation and 95% CI observed was 5.40 µM and 10.59 µM, respectively. The error as a percentage of the dynamic range did not exceed 10.84% for all experiments.

**Table 2:** The repeatability and reliability of experiments

Experiment	By tracer concentration (μM)		By %ID to eaCSF at 3 h	
	Standard deviation	95% CI	Standard deviation	95% CI
2NHP2	4.34	8.51 (8.13% of range)	4.12	4.66
3G1	4.45	8.72 (6.81% of range)	3.55	4.02
1NHP1	4.13	8.08 (8.57% of range)	1.00	0.86
3G2	3.26	6.40 (6.86% of range)	2.61	3.08
1NHP3	2.47	4.85 (5.18% of range)	0.58	0.65
1NHP2	5.40	10.59 (10.84% of range)	0.72	0.82
2G2	1.88	3.79 (3.56% of range)	1.13	1.02
5NHP1	2.72	5.33 (5.90% of range)	3.73	4.22

### Effect of injection parameters on tracer distribution over three hours

**Fig. 5:**
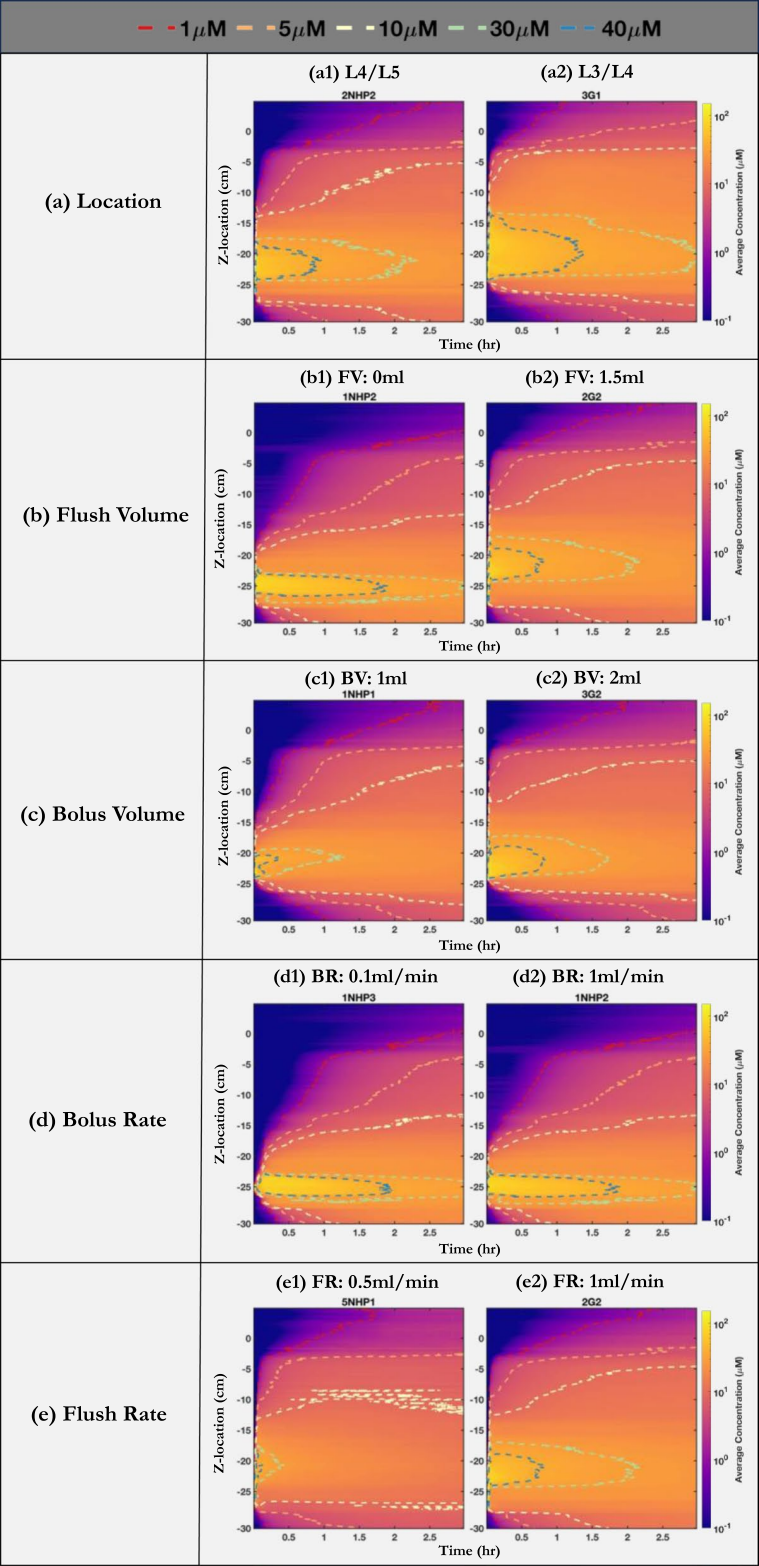
The axial distribution of tracer over three hours grouped by a change in (**a**) Location (**b**) Flush volume (**c**) Bolus volume (**d**) Bolus rate (**e**) Flush rate. The spatial–temporal plots of the average concentration when (**a1**, **a2**) injection location is at L4/L5 and L3/L4, respectively (**b1**, **b2**) flush volume is 0 ml and 1.5 ml, respectively (**c1**, **c2**) bolus volume is 1 ml and 2 ml, respectively (**d1**, **d2**) bolus rate is 0.1 ml/min and 1 ml/min, respectively (**e1**, **e2**) flush rate is 0.5 ml/min and 1 ml/min, respectively. The dashed lines represent concentration profiles at 1 µM (red), 5 µM (orange), 10 µM (yellow), 30 µM (green), and 40 µM (blue). *FV: flush volume, FR: flush rate, BV: bolus volume, BR: bolus rate*

Figure 5 compares the spatial–temporal distribution of the tracer over three hours post-injection, grouped by the change in parameters between experiments. When the injection location is moved by one vertebrae level closer towards the brain i.e., from L4/L5 to L3/L4 (2NHP2-3G1), we observed increased spread in the cranial direction with higher tracer concentration in the cervical region (Fig. 5a2). The impact of flush volume was noticeable at the injection site, where the tracer appeared to move toward the brain (Fig. 5b2). The use of a 1.5 ml flush volume in 2G2 slightly increased the spread toward the brain. Similarly, when the bolus volume was increased from 1 to 2 ml (1NHP1-3G2), a higher tracer concentration was observed in the brain (Fig. 5c2). Increasing the bolus rate from 0.1 ml/min to 1 ml/min (1NHP3-1NHP2) and flush rate from 0.5 ml/min to 1 ml/min (5NHP1-2G2) showed no significant differences (Fig. 5d, e).

### Effect of injection parameters on %ID to eaCSF at three hours

The calculated %ID to eaCSF at three hours for each injection protocol is listed in Table 3. The variation in %ID (Δ%ID) resulting from changes in injection parameters is listed in Table 4. Among the parameters investigated in this study, injection location was found to be the most important factor that significantly improved %ID to eaCSF. Moving the injection location from L4/L5 to L3/L4 (2NHP2-3G1) increased % ID to eaCSF by 10.5%, and this effect was statistically significant with a p-value of 0.0282. Increasing flush volume from 0 ml to 1.5 ml (1NHP2-2G2) improved the tracer dispersion to eaCSF by 6.5%, which is also statistically significant (p = 0.0218). Increasing the bolus volume from 1 to 2 ml (1NHP1-3G2) increased the %ID to eaCSF by 2.3%, but this impact was not statistically significant (p = 0.1910). Increasing the bolus rate or flush rate had a negative impact on the tracer dispersion to eaCSF. When the bolus rate was increased from 0.1 ml/min to 1 ml/min (1NHP3-1NHP2), the %ID to eaCSF decreased by 0.2% (p = 0.5283). A 0.4% (p = 0.910) decrease was observed when the flush rate was increased from 0.5 ml/min to 1 ml/min.

**Table 3:** The %ID and the average AUC at three hours for all experiments

Experiment	%ID to eaCSF at 3 h	Avg AUC_0–3 h_ to eaCSF μM-hr
2NHP2	8.2	2.8
3G1	18.8	8.1
1NHP1	7.2	2.3
3G2	9.5	3.6
1NHP3	3.0	0.9
1NHP2	2.8	0.8
2G2	9.3	3.5
5NHP1	9.7	3.8

**Table 4:** Parametric comparison in terms of change in %ID to eaCSF at three hours

Parameter	Protocols	Range	%ID to eaCSF at 3 h	Δ%ID	*P* value
Location	2NHP2 vs. 3G1	L4/L5 vs. 3/L4	8.2 vs. 18.7	+ 10.5%	0.0282
Flush volume	1NHP2 vs. 2G2	0 vs. 1.5 (ml)	2.8 vs. 9.3	+ 6.5%	0.0218
Bolus volume	1NHP1 vs. 3G2	1 vs. 2 (ml)	7.2 vs. 9.5	+ 2.3%	0.1910
Bolus rate	1NHP3 vs. 1NHP2	0.1 vs. 1 (ml/min)	3.0 vs. 2.8	− 0.2%	0.5283
Flush rate	5NHP1 vs. 2G2	0.5 vs. 1 (ml/min)	9.7 vs.9.3	− 0.4%	0.910

### Effect of injection parameters on AUC trends

**Fig. 6:**
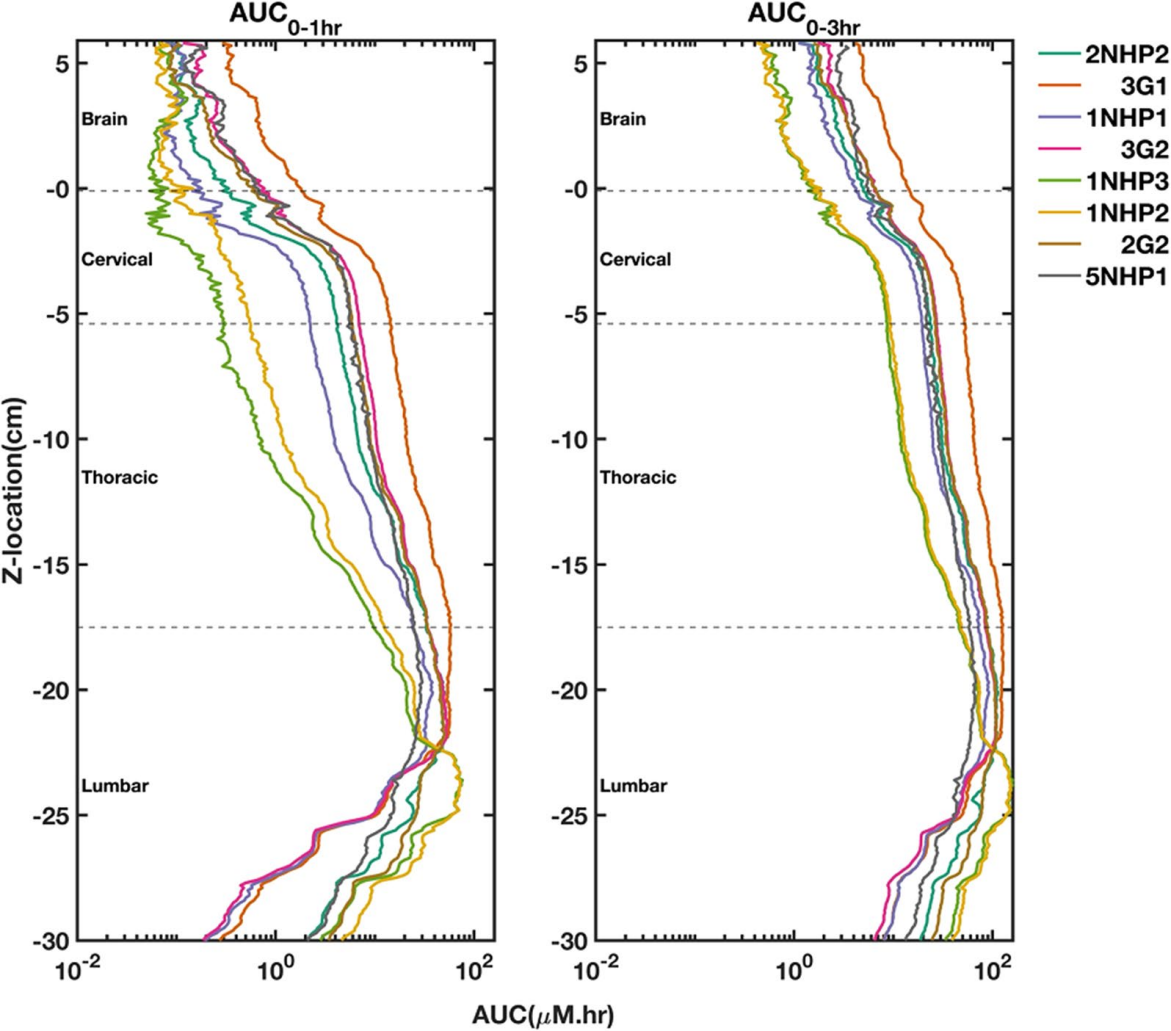
The area under the curve (AUC) profiles over one and three hours for all experiments

The AUC_0–1 h_ and AUC_0–3 h_ profiles are shown in Fig. 6. The AUC values were highest in the lumbar region and decreased towards the cranial directions in all experiments. The differences in AUC values across experiments tend to decrease over time. Regardless of the time, the 3G1 profile had the highest AUC values in the thoracic, cervical, and brain regions. The average AUC_0–3 h_ in the brain for each protocol is listed in Table 3. Moving the injection location from L4/L5 to L3/L4 (2NHP2-3G1) increased the average AUC_0–3 h_ in the brain by 5.3 µM-hr. Adding a flush volume of 1.5 ml improved the average AUC_0–3 h_ in the brain by 2.6 µM-hr (1NHP2-2G2). Increasing the bolus volume from 1 to 2 ml (1NHP1-3G2) increased the average AUC_0–3 h_ in the brain by 1.4 µM-hr. However, changes in the bolus or flush rates resulted in a decrease of 0.1 µM-hr in the average AUC_0–3 h_ to the brain.

## Discussion

Across the range of parameters investigated for lumbar puncture-based intrathecal injections, injection location had the biggest impact on increasing the %ID to eaCSF at 3 h, followed by flush volume and bolus volume. Whereas increasing bolus rate and flush rate had a slightly negative impact on %ID to eaCSF (Table 4). The change in %ID to eaCSF due to (a) injection location, (b) flush volume, (c) bolus volume, (d) bolus rate, and (e) flush rate ranged from + 10.5% to − 0.4%. Given the controlled boundary conditions and a single subject-specific CSF geometry, the observed variations in tracer distribution to the brain can be ascribed to injection parameters only.

### Impact of injection location on %ID to eaCSF

We examined the impact of lumbar injection sites on solute distribution by considering the two most common lumbar puncture sites typically used in pre-clinical NHP studies. Injections at the L3/L4 (3G1) site resulted in a + 10.5% enhancement in %ID to eaCSF at three hours compared to those at the L4/L5 (2NHP2) site. The tracer injected at L3/L4 (3G1) appeared to move to a greater degree cranially than caudally (Fig. 5a). This could be due to steady streaming flow patterns induced by the shape of nerve roots, spinal curvature, and changes in the cross-sectional area of the subarachnoid space [38]. Throughout all experiments conducted in this study, the model was mounted in the left lateral recumbent position. However, altering to the Trendelenburg position after lumbar injection could potentially impact the distribution of solutes to the brain [39]. Additionally, the shorter anatomical distance from L3/L4 to the brain compared to that from L4/L5 also appears to play a part in this increase in %ID. An in vivo PET imaging study by Papisov et al. [22] revealed that about 50% of the injected volume was delivered to cisterna magna in about thirty minutes when ~ 3.5 ml was injected at the L1 location in NHP. This observation emphasizes the role of both the cranially directed CSF flow dynamics in the subarachnoid space and the anatomical proximity to the brain in influencing solute distribution.

### Impact of flush volume on %ID to eaCSF

Though the L3/L4(3G1) location improved solute delivery to eaCSF, largely due to its nearer location to the brain than L4/L5(2NHP2), both protocols involved a bolus proceeded by a flushing process. To understand the sole effect of the flush volume, we contrasted the 1NHP2 and 2G2 protocols executed at the L4/L5 site, but one without and the other with a flush. At three hours post-injection, we noticed an increase of + 6.5%ID to eaCSF. The 2G2 procedure, involving the flush, displaced the tracer cranially, making a more rapid arrival at the foramen magnum (z-location: 0 cm) in contrast to the flush-free 1NHP2 approach (Fig. 5b). A Similar trend was reported by Wolf et al. [40] in their dual isotope imaging study on rats. In their experiment, rapid arrival of radiotracer at the foramen magnum was seen when the bolus was followed by a flush. While there are clear interspecies differences, the trends are comparable due to the similarity in the proportion of total volume (sum of bolus and flush) injected relative to CSF volume. The rat study involved an injection of ~ 12.5% of their CSF volume, in comparison to ~ 15% of the total CSF volume injected in our study.

### Impact of bolus volume and rate on %ID to eaCSF

In our findings, we observed that an increase in bolus volume led to increased distribution of solute to the brain, registering a + 2.4%ID to eaCSF at three hours (Fig. 5c). This is consistent with prior research showing that an increase in bolus volume can improve rostral distribution [24, 30]. For instance, Sullivan et al. [24] conducted an imaging investigation using NHPs and found that larger bolus volumes were more effective in transporting tracer to the brain, regardless of the specific infusate used. Additionally, a PET study by Tangen et al. [30] also showed this observation, noting that using a larger bolus of 1.8 ml led to the presence of radiotracer in the cranial region within two hours, whereas the 0.36 ml bolus resulted in a distribution more confined near the lumbar puncture injection site. In addition to the above observations, we evaluated the effect of bolus rate and flush rate on tracer distribution. Despite increasing the bolus rate and flush rate by ten-fold and two-fold respectively, the tracer distribution remained nearly unaffected (Fig. 5d,e). However, there was a minor decrease in the %ID to eaCSF by 0.2% and 0.4% respectively, which, upon statistical evaluation was found to be insignificant.

### Limitations

The in-vitro modeling in this study has several limitations. One significant restriction of this investigation was the inability of our model to evaluate the penetration of solutes into the CNS tissues. Hence our aim was to visualize and quantify early-stage pharmacokinetics of solute dispersion shortly after injection within the lumbar spine. We used a generalized model of solute dispersion with an aqueous solution of fluorescein serving as a small molecule drug surrogate. Drug-specific diffusion, which can impact the dispersion rate, was not accounted for in this study. Additionally, the model used in this study was rigid and both the spinal and intracranial compliance were not considered. Furthermore, this study was based on the CSF geometry of a single subject. The variability across geometric and hydrodynamic parameters, as characterized among NHPs by Khani et al. [31], could potentially influence solute distribution. Further research is required to quantify the impact of variables such as NHP size, age, and sex on the intrathecal transport. All our experiments were conducted using a single idealized CSF flow waveform for parametric comparison of injection-related parameters. However, prior studies indicate that both the amplitude and frequency of CSF flow, impact the intrathecal solute dispersion [25, 26]. Moreover, this study did not evaluate the effect of disease states which can alter CSF flow dynamics such as injuries, spinal stenosis, Chiari malformation, and syringomyelia. Due to the resolution limitations of 3D printing, microscopic anatomic structures, such as arachnoid trabeculae, were not included in our model geometry. These microanatomical structures may alter the CSF flow field [41], thereby changing solute dispersion. Lastly, this study has not directly validated the subject-specific intrathecal solute transport using in vivo data.

## Conclusions

We evaluated the influence of lumbar puncture injection parameters on intrathecal solute dispersion within a CSF system of an animal-specific 3D in vitro cynomolgus monkey. The lumbar injection parameters included: injection location, bolus volume and rate, and flush volume and rate. Our results indicate that changes to these parameters alter the distribution of solute along the neuro-axis, resulting in changes ranging from + 10.5%ID to − 0.4%ID to eaCSF at three hours post-injection. For the range of parameters evaluated, injection location significantly enhanced the solute delivery to eaCSF by + 10.5%ID (p = 0.0282). This was followed by the flush volume (+ 6.5%ID, p = 0.0218), and bolus volume (+ 2.3%ID, p = 0.1910). Conversely, both bolus and flush rates demonstrated minimal and statistically non-significant effects on solute distribution to eaCSF, with changes of − 0.2%ID (p = 0.5283) and − 0.4%ID (p = 0.910) respectively. Overall, our findings reveal that lumbar puncture injection parameters can be optimized to potentially improve brain biodistribution in NHP studies. However, for the injection parameters analyzed in this study, the maximum improvement effect size was limited to ~  + 10%. Future studies can be conducted to further improve algorithmic dosing procedures and injection devices and determine what maximum effect size is achievable.

## Data Availability

The datasets used and/or analyzed during the current study are available from the University of Idaho (DS), on reasonable request.

## Abbreviations

AUC: Area under the curve
BBB: Blood brain barrier
CI: Confidence interval
CNS: Central nervous system
CSF: Cerebrospinal fluid
DR: Dynamic range
eaCSF: Extra-axial cerebrospinal fluid
ID: Injected dose
IT: Intrathecal
MRI: Magnetic resonance imaging
PCMRI: Phase contrast magnetic resonance imaging
PET: Positron emission tomography
SAS: Subarachnoid space

## List of symbols

$${C}_{max}$$: Maximum concentration
$C(z)$: Concentration at slice-z
$V(z)$: Volume of slice-z
